# Morphology-Aware Prognostic Model for Five-Year Survival Prediction in Colorectal Cancer from H&E Whole-Slide Images: A Study Using Multi-Center Clinical Trial Cohort

**DOI:** 10.3390/cancers18071150

**Published:** 2026-04-02

**Authors:** Usama Sajjad, Abdul Rehman Akbar, Ziyu Su, Alejandro Leyva, Deborah Knight, Wendy L. Frankel, Metin N. Gurcan, Wei Chen, Muhammad Khalid Khan Niazi

**Affiliations:** 1 Department of Pathology, The Ohio State University Wexner Medical Center, Columbus, OH 43210, USA; 2 Center for Artificial Intelligence Research, School of Medicine, Wake Forest University, Winston-Salem, NC 27101, USA

**Keywords:** colorectal cancer, foundation models, multi-center survival prediction, deep learning

## Abstract

**Background:** Colorectal cancer (CRC) remains the third most prevalent malignancy globally, with approximately 154,000 new cases and 54,000 projected deaths anticipated for 2025. The recent advancement of foundation models in computational pathology has been largely propelled by task-agnostic methodologies that overlook organ-specific crucial morphological patterns that represent distinct biological processes that fundamentally influence tumor behavior, therapeutic response, and outcomes. **Methods:** In this study, we develop a novel, interpretable AI model, PRISM (Prognostic Representation of Integrated Spatial Morphology), that incorporates a continuous variability spectrum within each distinct morphology to reflect the principle that malignant transformation occurs through incremental evolutionary processes. PRISM is trained on 15 million histological images extracted from surgical resection specimens of 2957 patients. **Results:** PRISM achieved superior prognostic performance for five-year OS (AUC = 0.70 ± 0.04; accuracy = 68.37% ± 4.75%; HR = 3.21, 95% CI = 2.18–4.72; *p* < 0.0001 using multi-variate cox-proportional hazards model), outperforming existing CRC-specific methods by 15% and AI foundation models by ~23% accuracy. It showed sex-agnostic robustness (AUC Δ = 0.02; accuracy Δ = 0.15%) and stable performance across clinicopathological subgroups, with minimal accuracy fluctuation (Δ = 1.44%) between 5FU/LV and CPT-11/5FU/LV regimens, replicating the Alliance cohort finding of no survival difference between treatments. **Conclusions:** These results establish PRISM as a promising, interpretable tool for AI-driven prognostication, with potential for future extension to other cancer types and stages

## Simple Summary

Colorectal cancer (CRC) poses a significant global health burden, with over 154,000 new cases projected for 2025. Accurate survival prediction remains a critical challenge, as current pathological assessments fail to capture the full spectrum of morphological heterogeneity relevant to patient outcomes. This study presents PRISM (Prognostic Representation of Integrated Spatial Morphology), a novel AI model designed for five-year overall survival prediction in stage III CRC from routine H&E whole-slide images. PRISM integrates morphology-informed features derived from expert-annotated tissue regions with foundation model embeddings, enabling a biologically meaningful representation. PRISM is trained on over 15 million histological patches from 2957 patients, and achieved an AUC of 0.70 and an accuracy of 68.37%, outperforming existing CRC-specific and general-purpose AI models. We also demonstrated robust performance across sex, treatment regimen, and clinicopathological subgroups, representing a step toward interpretable AI-driven prognostication in CRC.

## 1. Introduction

Colorectal cancer (CRC) is the third most widespread cancer, with approximately 154,000 new cases and 54,000 deaths estimated for 2025 [1]. This epidemiological burden is characterized by pronounced stage-dependent prognostic stratification, with 5-year survival rates of 94.7% for stage I, 88.4% for stage II, 74.3% for stage III, and 31.5% for stage IV [2]. CRC is morphologically heterogeneous, and current pathological assessment does not capture the morphological variability in a quantitative manner and lacks multi-variate (multi-feature) assessments. For example, there are associations between grade, necrosis, and stroma with CRC prognosis [3,4,5,6], illustrating that these underutilized morphological features, if analyzed together through a more quantitative and multi-variate approach, could provide deeper insights into patient outcomes and improve prognostic accuracy. Recently, Tumor budding (TB) has been recognized as a marker of epithelial–mesenchymal transition and linked to poor prognosis [7,8,9]. Also, it has also been shown that necrosis is an independent prognostic variable with respect to progression-free survival [3]. Finally, stroma-rich tumors and those with immature desmoplastic stroma are also associated with worse outcomes [10]. These findings further underscore the prognostic value embedded in morphological characteristics, yet these morphological features remain underutilized in routine clinical assessments due to the lack of scalable, quantitative, and multi-variate analysis tools.

To address this gap, artificial intelligence (AI) systems have gained prominence, particularly with the digitization of tissue specimens into whole-slide images (WSIs) [11,12]. These technologies demonstrate robust performance in tumor classification, tumor subtyping, gene expression profiling, and automated analysis of nuclear/cellular features [12,13,14,15,16,17,18,19]. More recently, foundation models trained using self-supervised learning on millions of WSIs have emerged as a dominant paradigm in computational pathology [20,21,22,23,24,25]. These models learn to identify generic visual patterns by minimizing feature distances between augmented versions of the same image (e.g., through image crops, color or rotation changes) [26]. However, this domain-agnostic training approach presents a critical limitation for CRC prognostication: it often overlooks organ-specific morphological features that reflect distinct biological processes and are essential for predicting tumor behavior, treatment response, and patient outcomes. As a result, despite their generalizability, these models may fall short in capturing the nuanced, morphology-driven prognostic signals crucial for CRC.

Given the morphological heterogeneity of CRC [27], a tool that fails to incorporate morphologically relevant heterogeneous features would be unable to adequately quantify and analyze the variability within distinct morphological regions [28]. While existing computational tools such as QuantCRC [28] have made significant contributions to CRC prognostication, they primarily focus on discrete quantification of morphological features without fully capturing the continuous spectrum of phenotypic variability present within distinct histological microenvironments within WSIs. QuantCRC’s prognostic performance was evaluated using the concordance index, with inter-group differences assessed via log-rank test, while Kather et al. [29] trained a morphology classifier on NCT-HE-100K [30] and evaluated the prognostic contribution of each morphological class through max-pooled scores using univariable Cox proportional hazard models. Similarly, Nakanishi et al. [31] framed recurrence prediction as a binary classification problem, stratifying patients into recurred and non-recurred groups, with model performance assessed using classification metrics and statistical significance established through Mann–Whitney U-test and chi-squared tests. Therefore, an AI prognostic model is needed that integrates the diverse variability spectrum within each distinct morphology for prognostication.

**Figure 1:**
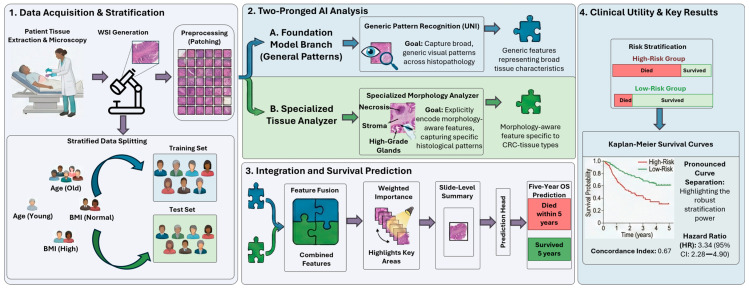
PRISM: An end-to-end AI framework that transforms whole-slide images (WSIs) into integrated prognostic signatures. (**1**) **Data acquisition and stratification:** Tissue specimens are surgically resected, formalin-fixed, stained with H&E, and digitally scanned. Patient data are partitioned into training and test sets, and all WSIs undergo preprocessing to extract fixed-size patches. (**2**) **Two-pronged AI analysis:** PRISM comprises two parallel branches: (i) a foundation model branch that extracts generic histopathological features, and (ii) a specialized morphology classifier (Specialized Tissue Analyzer) that extracts morphology-aware features. (**3**) **Integration and Survival Prediction:** The outputs of both branches are integrated to highlight prognostically relevant tissue regions. (**4**) **Clinical Utility and Key Results:** This combined representation enables precise five-year overall survival (OS) prediction and stratification of patients into distinct clinical risk groups, visualized through Kaplan–Meier survival curves.

To address these limitations, we propose PRISM (Prognostic Representation of Integrated Spatial Morphology), an AI model that integrates CRC morphologically relevant heterogeneous features for five-year overall survival (OS) prognostication (Figure 1). PRISM advances beyond binary morphological detection and quantification by incorporating a continuous variability spectrum that characterizes the phenotypic diversity within each distinct morphological pattern. This approach captures subtle gradations in cellular architecture and tissue organization that exist within nominally similar morphological categories. Unlike pathologists who may label a region simply as “cancer,” PRISM goes further by capturing the nuanced variations among neoplastic cells, for example, differences in nuclear morphology, gland formation, and architectural patterns, and leverages this detailed phenotypic diversity to enhance prognostication. PRISM captures biologically relevant information through a domain-specific branch and complements it with generic histopathological features extracted through a parallel branch, enabling a comprehensive and nuanced prognostic representation. This precise modeling of survival outcomes may enable more informed therapeutic decision-making, personalized treatment stratification, and improved resource allocation in clinical settings (Figure 1). Our study also reveals a fundamental limitation in AI models development for prognosis: Conventional validation strategies such as K-fold cross-validation are demonstrably inadequate for robustly evaluating OS prediction models in histopathology. PRISM incorporates a comprehensive strategy that integrates clinical and pathological attributes prior to training and evaluation to minimize confounding effects.

## 2. Datasets and Preprocessing

### 2.1. Ethics Statement and Patient Cohorts

For our main analysis, we used data from Cancer and Leukemia Group B (CALGB) 89803, a randomized phase III trial that compared adjuvant weekly bolus IFL versus FL alone in 1264 patients with completely resected stage III CRC (Identifier: NCT00003835 (CALGB 89803); **Registry:** CTRP (Clinical Trial Reporting Program); Registry Identifier: NCI-2012-01844; First Submitted: 1 November 1999; First Posted: 27 April 2004; Study Start: May 1999). CALGB is now part of the Alliance for Clinical Trials in Oncology, so this is referred to as the Alliance cohort. Eligible patients had no prior chemotherapy/radiotherapy history, performance status 0–2, and initiated treatment within 21–56 days post-resection. Stratification factors included lymph node involvement (1–3 vs. ≥4 nodes), histology grade, and preoperative CEA. The FL arm received the Roswell Park regimen (Leucovorin (LV) 500 mg/m^2^ + 5-Fluorouracil (5FU) 500 mg/m^2^ weekly × 6, every 8 weeks for four cycles). The IFL arm received Irinotecan (CPT-11) 125 mg/m^2^ + LV 20 mg/m^2^ + 5FU 500 mg/m^2^ weekly × 4, every 6 weeks for 5 cycles. Primary endpoints were overall survival (OS) and disease-free survival (DFS). Sample size and power calculations were based on OS, defined as the time from treatment initiation to death from any cause. A total accrual target of 1260 patients was set to achieve 82% power to detect an improvement in median survival from 8.0 years with FU plus LV alone to 10.5 years with CPT-11 plus FU plus LV (hazard ratio [HR] = 1.3; one-sided log-rank test, α = 0.05). The trial was approved by institutional review boards at all participating centers in cooperative groups CALGB, NCCTG, NCIC CTG, ECOG, SWOG, and all patients provided written informed consent. We have provided the detailed characteristics of the dataset in Table 1.

### 2.2. Tissue Scanning and Quality Assurance Protocol

All tissue samples in the Alliance cohort were resected from one of the following tumor locations: cecum, ascending colon, hepatic flexure, transverse colon, splenic flexure, descending colon, or sigmoid colon. Following resection, all samples underwent formalin fixation and were subsequently stained with H&E. WSIs of Alliance cohort were then acquired through digital scanning at 40× magnification (0.25 μm/pixel resolution) using an Aperio Digital Pathology Scanning system (Leica Biosystems, Nussloch, Germany) at The Ohio State University, Wexner Medical Center, Columbus, Ohio. This study received approval from the Ohio State University Institutional Review Board (IRB 2018C0098) with a waiver for informed consent. These WSIs were individually, manually reviewed by a trained expert pathologist to ensure that a tumor was present. Over >98% of cases had one representative tumor slide/WSI for evaluation. Exclusion criteria included: WSI with no tumor, lymph node tissue only, or mucinous (tumor composed of pools of mucin with floating tumor cells only) cases, censored within five years, and those with death attributed to causes other than disease from within the five-years deceased group. Our decision to exclude rather than censor these patients was driven by our specific research objective: to develop a prognostic model for cancer-specific mortality rather than all-cause mortality. In CRC, the distinction between these endpoints is clinically meaningful, particularly given that median age at diagnosis is 66 years [32] and many patients have competing comorbidities. Cases were also reviewed for quality check to ensure correct tissue detection and coverage, color fidelity, focus/sharpness, and no scanning artifacts; WSIs with inadequate staining were also removed. This comprehensive quality control process resulted in the exclusion of 211 cases from the original Alliance cohort, leaving 424 cases for PRISM training and evaluation. Among these 424 cases, patients with an overall survival time of less than five years were considered deceased, while the remaining patients were designated as survivors.

Throughout the entire scanning and tissue quality assessment process, reviewing pathologists remained blinded to all patient clinical information and outcomes. This rigorous filtering ensures that the dataset used for training PRISM is high-quality, relevant, and consistent, which is crucial for reliable prognostication.

## 3. Methods

### 3.1. Identification of Subgroups with Varied Survival Rates and Stratified Data Splitting for Effective Generalization

A critical aspect of developing robust prognostic models involves understanding the heterogeneity within patient populations and ensuring that model training and evaluation account for clinically relevant subgroups. Traditional approaches to model validation often overlook the inherent diversity in patient demographics and clinical characteristics, which can lead to biased performance estimates and reduced generalizability across different patient populations [33]. To address this fundamental limitation, we conducted a comprehensive analysis of patient subgroups within our Alliance cohort to identify distinct populations with varying survival outcomes. We then implemented a stratified approach to data splitting that preserves the representation of these diverse patient characteristics.

**Figure 2:**
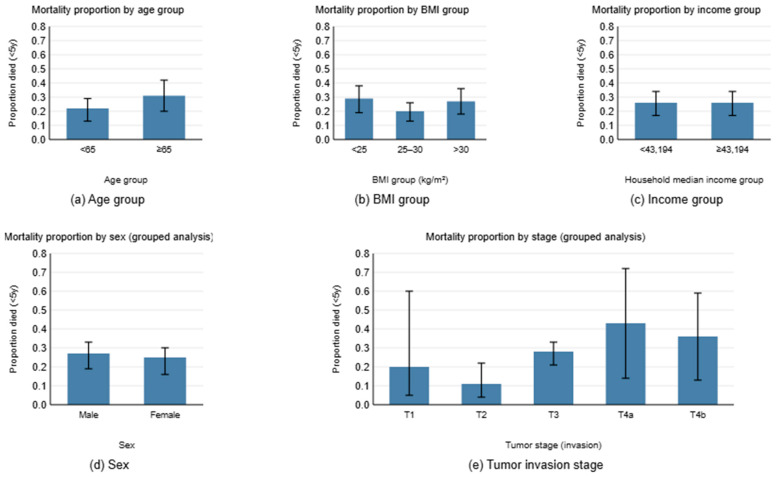
Association between clinical variables and 5-year mortality. (**a**) Age grouped as <65 vs. ≥65 years. (**b**) BMI grouped as <24, 25–30, and >30 $kg/m^{2}$. (**c**) Household income grouped at $43,194. (**d**) Sex. (**e**) Tumor invasion stage (T1–T4b). Bars show the proportion of patients who died within 5 years in each category, with 95% confidence intervals.

Our quantitative analysis of clinical data revealed distinct patient subgroups with different survival outcomes across multiple demographic and clinical parameters (Figure 2). We systematically grouped patients based on several key criteria and assessed five-year survival rates within each subgroup. Age stratification demonstrated significant prognostic value, with patients aged ≤65 years exhibiting substantially different five-year survival rates compared with those >65 years. This age-based survival disparity reflects the complex interplay between chronological age, comorbidity burden, treatment tolerance, and overall physiological reserve in CRC patients. Similarly, we identified survival variations based on socioeconomic factors, particularly income levels, which may serve as a proxy for healthcare access, treatment compliance, and overall health status. Income-based stratification can reveal patterns that are likely to reflect differences in healthcare quality, early detection rates, and access to optimal treatment regimens. For example, lower-income patients frequently encounter systemic barriers including inadequate insurance coverage, limited access to specialist services, and pronounced financial constraints that collectively impede adherence to guideline-concordant care. These disparities further extend to early detection, as individuals from higher-income groups demonstrate greater utilization of recommended cancer screening examinations, such as colonoscopy, facilitating diagnosis at earlier and more therapeutically amenable disease stages. Compounding these inequities, access to optimal personalized treatment remains inequitably distributed, as costly targeted therapies and immunotherapeutic agents including immune checkpoint inhibitors are disproportionately available to patients with greater financial resources or comprehensive insurance coverage, with direct implications for survival outcomes. Body mass index grouping also demonstrated distinct patterns, with patients categorized into three groups including BMI < 25 kg per square meter, BMI 25 to 30 kg per square meter, and BMI ≥ 30 kg per square meter. These BMI-based subgroups showed differences that may relate to nutritional status, metabolic health, treatment toxicity, and surgical outcomes in CRC patients. Age demonstrated a borderline trend, with higher mortality in patients ≥65 years compared with <65 years, with observed rates of 31.8 percent and 22.7 percent respectively, with a chi squared value of 3.36 and a *p* value of 0.0667. BMI category was not associated with mortality, with a chi squared value of 3.90 and a *p* value of 0.1422, nor was median household income, which showed a chi squared value of 0.00 and a *p* value of 1.000. Sex likewise showed no significant effect, with a chi squared value of 0.55 and a *p*-value of 0.460. Tumor invasion stage exhibited increasing mortality in advanced categories, although this trend was not statistically significant, with a chi squared value of 6.33 and a *p*-value of 0.176. These results indicate that while observable differences exist across demographic and clinical groups, none reached statistical significance in this dataset. The absence of strong univariable predictors indicates that prognosis in this cohort is not readily explained by conventional demographic or clinical factors. This pattern suggests that survival outcomes are influenced by more complex or subtle features that routine clinical variables do not capture. As a result, the value of our proposed model is strengthened, because its predictive performance reflects the identification of information that is not available through standard clinical measures. Instead of reproducing known clinical effects, the model appears to provide additional prognostic insight by detecting patterns that are not visible in traditional covariates.

**Figure 3:**
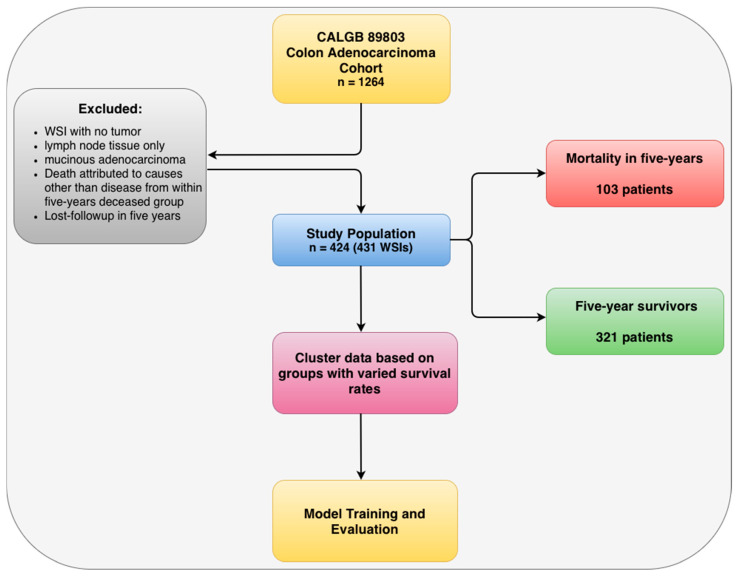
Study population and cohort design. A flowchart illustrating the patient selection process for colon carcinoma cases, including exclusion criteria: whole-slide images (WSIs) with no tumor, lymph node tissue only, mucinous adenocarcinoma, non-disease-related deaths within five years, and cases lost to follow-up within five years. The final cohort included 424 patients with 431 WSIs, stratified into deceased (*n* = 103) and survivor (*n* = 321) groups based on five-year follow-up. To ensure balanced representation across varying survival rate groups, patients were clustered based on age, BMI, and income, and five-folds were constructed within each cluster. These were then concatenated to form training, validation, and test sets.

To ensure robust model development and evaluation, we implemented a stratified data splitting approach that maintained proportional representation of these clinically relevant subgroups across training and validation sets (Figure 3). Specifically, we used K-modes clustering on age, income, BMI and sex based on the provided bins in Figure 2 specifically to create the splits only. As a result, PRISM prevents the inadvertent creation of training sets that over-represent or under-represent specific patient populations, thereby ensuring that PRISM’s prognostic performance is evaluated against the full spectrum of patient diversity present in clinical practice. This approach addresses the critical limitation of conventional validation strategies that may produce overly optimistic performance estimates by failing to account for population heterogeneity and potential subgroup-specific biases embedded within histopathological images.

### 3.2. Morphology-Informed Classifier and Feature Extractor

Current foundation models in computational pathology have demonstrated strong generalization across tasks [20,21], but they do not explicitly encode morphological semantics. These models typically rely on self-supervised learning to capture broad visual representations, often overlooking the domain-specific morphological features that are critical for accurate prognostication. In contrast, pathologists do recognize these patterns but tend to describe them in discrete terms, labeling regions as “neoplastic,” “stromal,” or “inflammatory” without accounting for the continuous phenotypic variation that exists within these categories. Existing computational systems that attempt to extract morphological features often follow a two-step process: first, they classify tissue regions into predefined categories; then, they compute basic statistics (e.g., area, density) within those identified regions. While useful, this approach treats morphology as static and compartmentalized, failing to capture the gradual transitions and phenotypic diversity that reflect tumor evolution and biological complexity.

PRISM addresses this critical gap by explicitly modeling the continuous morphological variability within and across histological regions. Rather than relying solely on categorical labels or generic feature extraction, PRISM learns to represent nuanced phenotypic differences such as subtle changes in nuclear morphology, glandular architecture, and stromal composition and integrates these with generic histopathological features to construct a more biologically faithful and prognostically powerful representation. To extract these morphology-informed features, we developed a deep learning model trained on the publicly available Histopathology AI (Hist-AI) colorectal dataset [34]. WSIs are first annotated by expert pathologists to identify 13 distinct morphological tissue regions, and histological patches are then extracted from these annotated regions to train a morphology classification module. The Hist-AI dataset contains 13 distinct tissue morphologies, including: High-Grade Adenocarcinoma, Low-Grade Adenocarcinoma, High-Grade Adenoma, Low-Grade Adenoma, Fat, Hyperplastic Polyp, Inflammation, Mucin, Smooth Muscle, Necrosis, Sessile Serrated Lesion, Stroma, and Vascular Structures. We used 224 × 224-pixel patches extracted at 20× magnification to train a morphology classification network.

**Figure 4:**
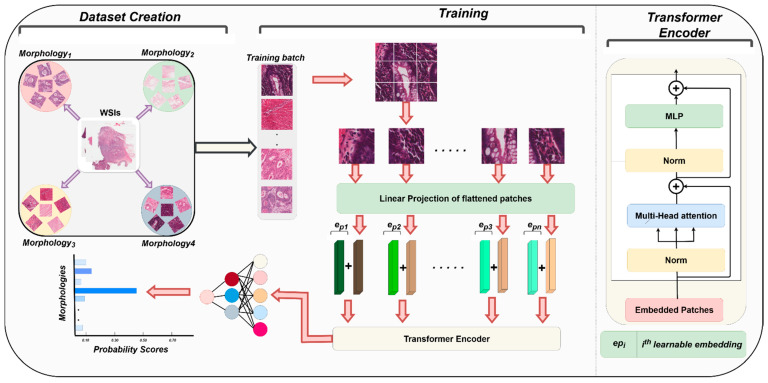
Overview of PRISM’s morphology classifier training pipeline. Whole-slide images (WSIs) are first annotated by expert pathologists to identify 13 distinct morphological tissue regions (Hist-AI dataset). Histological patches are then extracted from these annotated regions and used to train a morphology classification module. Once trained, this module generates specialized morphology-informed features, which PRISM integrates with generic histopathological features to construct a robust, multi-faceted representation for five-year survival prediction in stage III CRC.

During training, PRISM not only learns to classify these morphologies but also captures the intra-class variability within each category. This enables the model to extract a diverse set of high-dimensional features that reflect the phenotypic spectrum within each morphological type. The network was optimized using cross-entropy loss and evaluated using five-fold cross-validation to ensure generalization. For downstream prognostication, the final classification layer was removed, and the penultimate fully connected layer was used as a feature extraction backbone (Figure 4). These learned features rich in tissue-specific morphological information serve as the foundation for PRISM’s survival prediction model, enabling it to capture clinically relevant patterns that are often missed by traditional approaches.

### 3.3. Morphology-Aware Survival Prediction

Building upon the morphology-informed feature extraction capabilities described above, PRISM integrates these domain-specific features with foundation model embeddings to construct a comprehensive AI prognostic model. Foundation models [20], trained on large-scale histological datasets using self-supervised learning, capture broad visual patterns but lack explicit encoding of biologically meaningful morphological semantics. In contrast, PRISM’s morphology-informed features are derived from expert-annotated tissue regions and capture nuanced phenotypic variations such as differences in nuclear morphology, glandular architecture, and stromal composition that are critical for prognostication. By combining the generalizability of foundation models with the specificity of morphology-aware representations, PRISM creates a more biologically faithful and prognostically powerful feature space.

To effectively model survival outcomes, PRISM employs a multi-instance learning (MIL) paradigm [35], which accommodates the variable number of tissue patches per WSI and learns to weigh the relative importance of different morphological patterns. This approach addresses the critical need for prognostic models that can capture the complex interplay between diverse tissue morphologies while accounting for the inherent spatial and phenotypic heterogeneity present within CRC slides. The resulting model not only improves predictive performance but also enhances interpretability by linking prognostic signals to specific morphological contexts.

Here are the implementation details of PRISM. Let X = {$X_{1},\;X_{2},\;..,X_{n}$} represent the n patient WSIs with five-survival Y = {$y_{1},\;y_{2},\;..,y_{n}$}, where $y_{n}\in \{0,\;1\}$ for i={1,…, n} corresponds to the five-survival label with 1 means that patient died within five years and 0 means that patient survived for five-years post-resection. For each WSI $X_{i}$, for i={1,…, n}, we divide $X_{i}$ = {$x_{i1},\;x_{i2},\;..,x_{im}$} into m patches with each patch $x_{i1}$ representing 224 × 224 area at 20× magnification, and patches with less than 25% of the tissue are excluded using TRIDENT [36,37]. Then, a vision transformer-based histopathology trained feature extractor (UNI) [20,38] is used to extract the features $g_{ij}$ from each patch $x_{ij}$ ∈ $R^{d}$ of the WSI $X_{i}$. Subsequently, we also feed each patch $x_{ij}$ of the WSI $X_{i}$ from the morphology-informed feature extractor and extract the morphology-aware features $m_{ij}$ for each patch $x_{ij}$ ∈ $R^{d}$ of the WSI.

Once we extract both features ($g_{ij}$ and $m_{ij}$) from each patch $x_{ij}$, we use two learnable projections $W_{g}$ and $W_{m}$ for $g_{ij}$ and $m_{ij}$ and compute the cross-feature interaction of each morphology-aware feature with each foundation model feature to generate ${f^{\prime }}_{ij}$, where ${f^{\prime }}_{ij}$ ∈ $R^{d\;x\;d}\;$(Figure S1). Then, we use a neural network to project them again as $f_{ij}$ ∈ $R^{d}$ to merge the morphology-informed features with foundation model features. Specifically, we learn a transformation T: $R^{d\;x\;d}\to$ $R^{d}$ using the neural network that maximizes the mutual information I($f_{ij};\;{f^{\prime }}_{ij}$) = I ($f_{ij};\;T(f_{ij})$) while enforcing the constraint ${d\;x\;d}^{\prime }$ << d. Since each slide $X_{i}$ contains a different number of patches, and each patch may have varying prognostic significance, we employ multiple instance learning (MIL) aggregation [35] to combine the morphology-informed patch features and generate the slide-level representation $Z_{i}$ (Equation (1)),

(1) $$Z_{i}=\sum_{j=1}^{n}a_{ij}f_{ij}$$

where

(2) $$a_{ik}=\frac{exp(W^{T}\left(\tanh\left(V^{T}f_{ik}\right)\right)\;⊙\;V^{T}\left(\mathrm{sigm}\left(U^{T}f_{ik}\right)\right))}{\sum_{j=1}^{n}exp(W^{T}\left(\tanh\left(V^{T}f_{ij}\right)\right)\;⊙\;V^{T}\left(\mathrm{sigm}\left(U^{T}f_{ij}\right)\right))}\;\;\;\;\;\;\;\;\;\;\;\;$$

is the importance score $a_{ik}$ computed for each patch $x_{ik}$, which quantifies the relative prognostic relevance of patch $x_{ik}$ with respect to other patches within the WSI $X_{i}$ (Equation (2)). Here, V ∈ $R^{d\;x\;l}$, U ∈ $R^{d\;x\;l}$, and W ∈ $R^{\;l\;x\;1}$ are the learnable parameters of the attention network, l represents the number of neurons in the hidden layer, and ⊙ denotes element-wise multiplication. This morphology-informed slide representation $Z_{i}$ is subsequently used by a separate neural network to predict five-year OS (Figure S1). During the training process, all cross-modal interactions between generic histopathological features and morphology-informed features for each patch $x_{ij}$ are learned and optimized in an end-to-end fashion, which enables the optimization of feature combinations most relevant for prognostic prediction using a binary-cross-entropy loss function. Specifically, PRISM tries to optimize $W_{g},$ and $W_{m}$ parameters to extract task-specific features, while jointly learning optimal *W* and U parameters that minimize the loss, ultimately leading to attributing higher $a_{ik}$ scores to distinctive morphological regions among the subgroups.

### 3.4. Evaluation Protocols and Implementation Details

To train our morphology-informed feature extraction module, we utilized the publicly available Hist AI colorectal dataset [34]. This dataset comprises 77,182 annotated histological patches derived from 1719 H&E-stained WSIs, systematically categorized into 13 distinct morphological classes. For training, we used 70% of the histological patches from each morphological class, with 15% allocated for validation and the remaining 15% for testing. This stratified sampling approach ensured balanced representation across all tissue types in each data split.

For training PRISM, we preprocessed and partitioned each WSI into 224 × 224 × 3 patches at 20× magnification using TRIDENT [36,37] and excluded patches containing less than 25% tissue content. Then, we extracted generic histopathological features using a foundation model (UNI) [20] to ensure consistent feature representation across all comparative approaches [31,35,39,40,41]. Since UNI is pretrained on millions of histopathological images and inherently captures staining variability, explicit stain normalization was not applied as its intrinsic representational robustness sufficiently accounts for inter-institutional staining differences. For training the morphology-informed classifier, we implemented the Hibou foundation model [24] as a feature extractor, coupled with a multi-layer perceptron (MLP) head comprising two fully connected layers of 512, 128, and 13 dimensions, respectively, utilizing ReLU and SoftMax activation functions. Once the morphology-informed classifier was trained, we obtained morphology-informed features by truncating the model after the 512-dimensional layer to capture hidden feature representations. Subsequently, we configured PRISM training with the following hyperparameters optimized based on the validation set: learning rate of 2 × 10^−5^, Adam optimizer [42], Xavier uniform weight initialization [43], batch size of 1, and L1 regularization coefficient of 5 × 10^−4^. For comparative evaluation, we implemented baseline methods using default parameters from their respective GitHub repositories (accessed on 15 May 2025), with the exception of RRT-MIL [41], where we computed classification thresholds on the validation set to optimize performance evaluation using accuracy, AUC, sensitivity, and specificity (Equation (3)) [44]. We omitted dropout regularization as our model incorporated L1 regularization to promote feature sparsity and prevent overfitting to irrelevant morphological patterns. We employed Xavier uniform initialization [43] to ensure appropriate gradient flow and stable training dynamics throughout the deep architecture and maintained training reproducibility through fixed random seed implementation, enabling consistent results across multiple experimental iterations. For K-modes, we used K = 6 optimized using Elbow’s method.

(3) $$Accuracy=\frac{TP+TN}{TP+TN+FP+FN};\;Sensitivity=\frac{TP}{TP+FN};\;Specificity=\frac{TN}{TN+FP};\;\;\;\;\;$$

We evaluated the performance of PRISM on: (i) the Alliance cohort; and (ii) a publicly available dataset using The Cancer Genome Atlas (TCGA) CRC cohort, a multi-center study encompassing patients with stage I–IV disease, predominantly from institutions across the United States. All histopathological images and associated clinical data from the TCGA study are publicly accessible through the Genomic Data Commons portal (https://portal.gdc.cancer.gov).

For each cohort, we evaluated PRISM’s performance using five-fold cross-validation, with 20% cases allocated for testing (per-fold) from each cluster. From the remaining 80%, an additional 10% of the total cases was set aside for validation. To compare the statistical significance between methods, a two-sided Wilcoxon signed-rank test was applied to the sample-wise Brier scores, enabling a paired, non-parametric assessment of whether one model systematically achieves lower prediction error than the other across all samples. Kaplan–Meier curves were compared using the log-rank test.

## 4. Results

### 4.1. Deep Learning Predicts Colorectal Morphological Phenotypes and Enables Extraction of Morphology-Informed Features

The foundation of PRISM relies on accurate automated identification and quantification of distinct morphological phenotypes within colorectal histopathology. To establish this capability, we systematically validated our morphology-informed phenotyping on comprehensively annotated tissue patches from the HistAI colorectal dataset, ensuring robust performance across the diverse spectrum of morphological patterns encountered in CRC histopathology.

Our analysis demonstrated that training across this comprehensive spectrum of tissue morphologies yields a highly robust deep learning model for morphological phenotyping, achieving an overall accuracy of 90.43% and an AUC of 0.890. Critically, performance remained consistently high across all histological phenotypes, confirming the model’s capacity to resolve the intrinsic morphological heterogeneity characteristics of CRC histopathology. This high-fidelity morphological phenotyping indicates that the learned feature representations encode biologically meaningful histopathological patterns. These resulting morphologically derived features capture essential organizational principles within the tumor microenvironment, enabling automated quantification of key histomorphometry parameters, including tumor burden, stromal composition, vascular density, and the spatial distribution of prognostically significant morphological components.

### 4.2. Prognostic Stratification of Stage III CRC Patients Using a Morphology-Aware Deep Learning Network

To evaluate the prognostic accuracy of PRISM, we conducted a comprehensive evaluation on the Alliance cohort [45]. Our assessment focused on quantifying improvements in survival prediction accuracy while examining the model’s ability to maintain balanced performance across key clinical metrics. We compared PRISM against state-of-the-art multiple instance learning approaches (CLAM, TransMIL, ABMIL, RRT-MIL, Nakanishi et al.) [31,35,39,40,41] and existing CRC prognostication methods to establish its relative performance and clinical significance. Additionally, we conducted detailed analysis of sensitivity–specificity trade-offs and cross-validation stability to assess PRISM’s readiness.

**Figure 5:**
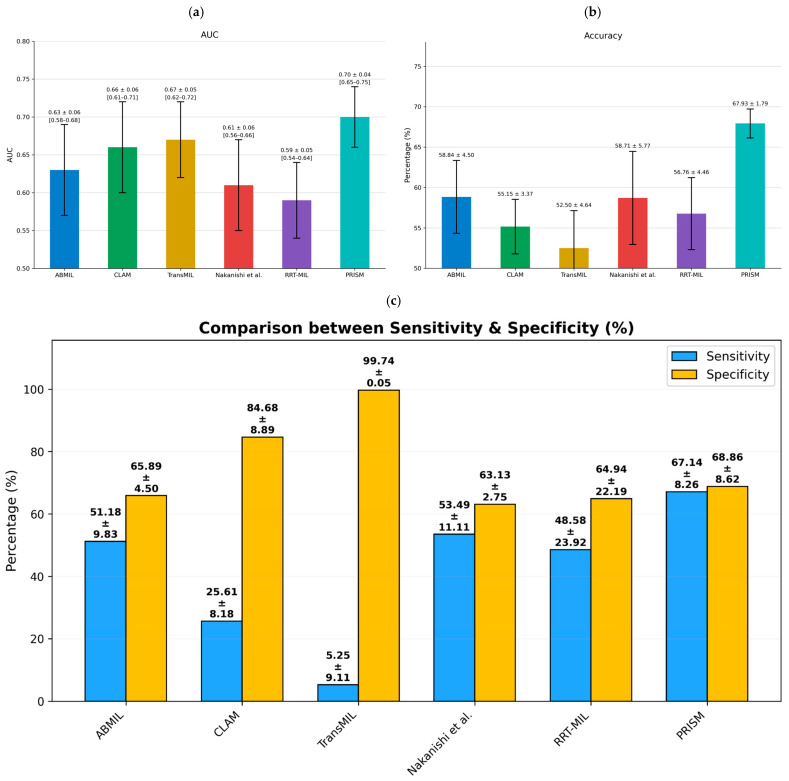
Five-year survival prediction results in the Alliance cohort using five-fold cross-validation. *X*-axis presents the results of different comparison methods: (**a**) Area Under the Curve (AUC) values with standard deviations and 95% CI; (**b**) accuracy percentages with standard deviations; and (**c**) grouped comparison of sensitivity and specificity with other models. Our model achieves the highest accuracy and balanced sensitivity and specificity, demonstrating superior performance compared to existing state-of-the-art methods (CLAM [39], TransMIL [40], ABMIL [35], RRT-MIL [41], Nakanishi et al. [31]).

First, we applied PRISM on the Alliance cohort (N = 424 patients), demonstrating significantly enhanced prognostic stratification for five-year post-resection survival (Figure 5). Overall, PRISM achieved an AUC of 0.70 ± 0.04 and an accuracy of 68.37% ± 4.75%, demonstrating >15% relative gain in accuracy over existing approaches (second-best (ABMIL): 58.84% ± 4.50%). We also compared PRISM with Nakanishi et al.’s [31] approach, which was proposed specifically for predicting CRC recurrence, and demonstrated a relative gain of 15%. Additionally, PRISM’s performance was statistically significant compared to all baseline methods using the two-sided Wilcoxon signed rank test (*p*-values: ABMIL = 0.012, CLAM = 0.018, TransMIL < 0.0001, Nakanishi et al. < 0.0001, RRTMIL < 0.0003).

We also evaluated PRISM’s performance using both sensitivity (accurate identification of patients who died within five years) and specificity (accurate detection of survivors at five years). As shown in Figure 5, PRISM achieved an optimal balance between these metrics, while comparison methods (CLAM [39], TransMIL [40], Ilse et al. [35], RRT-MIL [41], Nakanishi et al. [31]) exhibited 10–20% difference between sensitivity and specificity. While threshold adjustment could improve this balance for individual methods, it inevitably compromises the opposing metric. The consistently lower standard deviation of our model compared to alternatives demonstrates superior robustness, highlighting the importance of incorporating relevant histopathological features.

### 4.3. Comparison of Five-Year Survival Prediction Using PRISM Across Different Treatments

The potential impact of different chemotherapeutic regimens on tumor biology necessitates examining prognostic model performance across treatment-specific cohorts. While the original Alliance trial found no significant survival difference between FL and IFL treatment regimens, variations in model performance could indicate irrelevant learned features rather than intrinsic biological features.

We compared the performance of benchmark methods with PRISM by stratifying the treatment (FL vs. IFL). The results are presented in Table 2. PRISM demonstrated robust performance, with the highest AUC (0.7160 ± 0.061) and accuracy (68.21% ± 3.40), maintaining balanced sensitivity–specificity (64.82% ± 3.40/71.60% ± 8.50) on the Alliance cohort treated with FL only. In contrast, benchmark models exhibited critical limitations: TransMIL showed sensitivity collapse, and the high sensitivity and accuracy volatility of ABMIL. Nakanishi et al.’s approach [31], and RRT-MIL (±20.00 SD, ±11.43 SD) revealed fundamental instability in learning the morphologically meaningful features. Transitioning to the Alliance cohort treated with IFL, PRISM maintained a consistent accuracy (66.77% ± 5.10) and competitive AUC (0.6846 ± 0.108), with a degradation of ~0.030 AUC and 1.45% in accuracy across FL and IFL treatment groups, whereas benchmark methods displayed high performance degradation; ABMIL’s AUC decreased by ~7%, CLAM’s accuracy dropped 10%, Nakanishi et al.’s approach’s [31] accuracy differed by 5% across FL/IFL treatment groups, and RRT-MIL’s variability in accurately predicting patients surviving five years surged to ± 25.00 SD.

### 4.4. Morphology-Informed Features Correlation with Time-to-Event Survival

Beyond predicting five-year classification, we evaluated PRISM’s capacity for continuous risk stratification and survival prediction using established survival analysis metrics. This assessment provides critical insights into PRISM’s clinical utility for patient counseling and treatment planning decisions.

**Figure 6:**
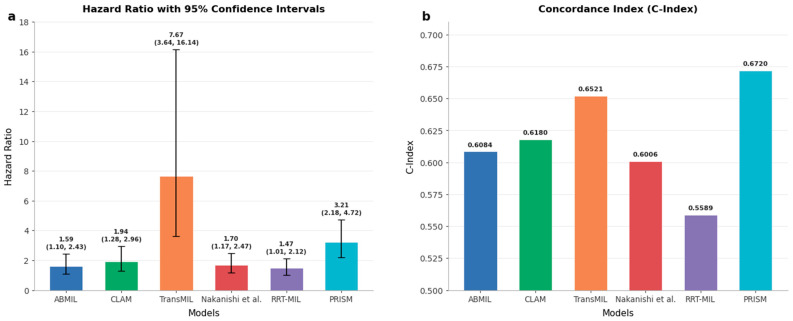
Hazard ratios and concordance index (c-index) values in the Alliance cohort using five-fold cross-validation. (**a**) Hazard ratios with 95% confidence intervals for each model and (**b**) concordance index (c-index) values demonstrating model ability to sort the patient based on risk for survival prediction. Our model achieves the highest c-index (0.6720) and comparable hazard ratio to TransMIL [40] with a smaller confidence interval, indicating superior prognostic performance compared to other state-of-the-art methods such as Nakanishi et al. [31].

**Figure 7:**
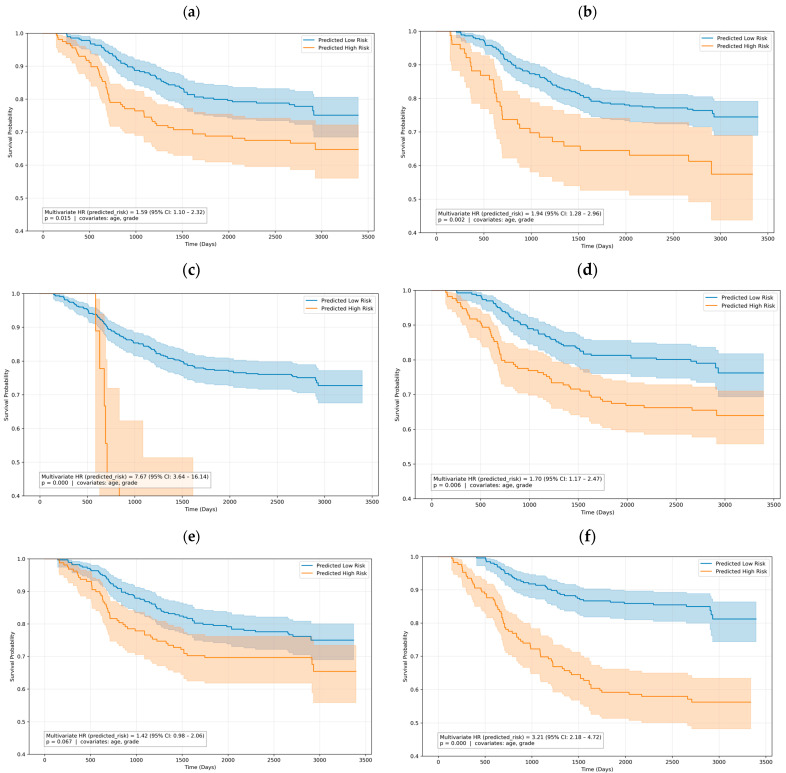
Kaplan–Meier plots for overall survival probability for five-year follow-up, with corresponding *p*-values (obtained using log-rank test) indicated on each plot. (**a**) ABMIL. (**b**) CLAM. (**c**) TransMIL. (**d**) Nakanishi et al. [31]. (**e**) RRT-MIL. (**f**) PRISM.

PRISM demonstrated remarkable performance in stratifying CRC patients into distinct prognostic risk categories using the multi-variate Cox proportional-hazards model, achieving a hazard ratio of 3.21 (95% CI: 2.18–4.72) and concordance index (c-index) of 0.67 (Figure 6; Supplementary Table S6). This represents a significant improvement over all benchmark methods: CLAM (HR: 1.94, 95% CI: 1.28–2.96; c-index: 0.62), Nakanishi et al. [31] (HR: 1.70, 95% CI: 1.17–2.47; c-index: 0.60), ABMIL (HR: 1.59, 95% CI: 1.10–2.32; c-index: 0.61), TransMIL (HR: 7.67, 95% CI: 3.64–16.14; c-index: 0.65), and RRT-MIL (HR: 1.42, 95% CI: 0.98–2.06; c-index: 0.56). While TransMIL showed a higher nominal hazard ratio, its broad confidence interval and reduced accuracy limits its ability for individualized risk stratification. Additionally, we visualized the Kaplan–Meier survival curves overall survival probability for five-year follow-up in Figure 7. PRISM achieved the highest hazard ratio of 3.34 (95% CI: 2.28–4.90) and a concordance index of 0.67. While benchmark methods such as CLAM [39] and Nakanishi et al. [31] show initial separation between risk groups, their curves demonstrate earlier convergence and less pronounced differences in survival probabilities, with ABMIL [35] displaying moderate separation but notable fluctuations suggesting less stable risk predictions, TransMIL [40] exhibiting erratic curve behavior with wide confidence intervals despite a high nominal hazard ratio (6.30), and RRT-MIL [41] demonstrating the poorest performance with minimal curve separation and the lowest hazard ratio (1.47).

## 5. Discussion

This study presents PRISM, a novel morphology-informed AI prognostic model that significantly advances the field of AI-driven prognostication in CRC. Our findings demonstrate that incorporating specific morphological features into deep learning models yields substantial improvements over conventional approaches, while simultaneously revealing critical limitations in current model validation practices and highlighting the importance of addressing algorithmic bias in clinical AI applications (Supplementary Materials, Sections S1.1–S1.3).

The superior performance of PRISM, achieving an AUC of 0.70 ± 0.04 and accuracy of 68.37% ± 4.75%, represents a meaningful advance over existing methodologies. The improvement in accuracy over the second-best-performing method translates to more precise risk stratification for approximately 1 in 10 patients, which could significantly impact clinical decision-making at population scales. More importantly, the hazard ratio of 3.21 (95% CI: 2.18–4.72) demonstrates robust discrimination between high- and low-risk patient populations, providing clinicians with actionable prognostic information that extends beyond traditional TNM staging [46]. In comparison with recently published prognostic models, PRISM demonstrates superior prognostic performance relative to recently published deep learning-based prognostic models in CRC. Specifically, automated TIL density assessment [47] achieved a univariate HR of 2.38 (95% CI: 1.57–3.61) and multivariate HR of 2.17 (95% CI: 1.42–3.33), while Stroma AReactive Invasion Front Areas (SARIFA)-based stratification [48] yielded HRs on two cohorts: 1.75 (95% CI 1.35–2.25), 2.09 (95% CI 1.43–3.05), whereas PRISM achieved a multivariate HR of 3.21 (95% CI: 2.18–4.72). Similarly, we evaluated PROGPATH [49] on our cohort, and obtained a c-index of 0.56, compared to a c-index of 0.67 achieved by PRISM, reflecting an absolute improvement of 0.11.

The success of PRISM validates the hypothesis that CRC’s inherent morphological heterogeneity contains prognostically relevant information that is inadequately captured by conventional histopathological evaluation. By explicitly training on the Hist-AI colorectal dataset [34] to recognize specific tissue morphologies that include the glandular carcinogenesis spectrum, serrated pathway precursors, tumor microenvironment characteristics, and invasion biomarkers, our model learns biologically meaningful features that align with established pathological knowledge. This targeted feature extraction addresses a fundamental limitation in foundation model pretraining, where self-supervised objectives are decoupled from the morphological patterns most relevant for prognostication. Additionally, a manual review conducted by an expert pathologist validated that high-attention regions within the deceased group corresponded to high-grade adenocarcinoma regions, lending histological credibility to the model’s outputs. In future studies, we will aim to leverage these findings toward developing a quantitative biomarker.

Our findings also reproduce the Alliance cohort clinical trial results, confirming no significant survival differences between treatment groups (5FU/LV versus CPT-11/5FU/LV). These treatment-driven performance differences highlight critical flaws in benchmark feature representations (Table 1). Despite clinical evidence of equivalent survival outcomes, models trained solely using foundation model-extracted features showed differences in AUC and accuracy, accompanied by high standard deviation. Such regimen-specific instability suggests that these models capture irrelevant features. PRISM’s minimal accuracy fluctuation (Δ = 1.44%) demonstrates alignment with the trial’s finding of no survival benefit differences between treatment regimens.

The balanced sensitivity (67.14% ± 8.26%) and specificity (68.86% ± 8.62%) achieved by PRISM addresses a critical challenge in clinical implementation, where extreme performance on one metric at the expense of the other can limit practical utility. Unlike benchmark methods that exhibited 10–15% differences between these metrics, our AI prognostic model provides reliable predictions across both outcomes in correctly identifying patients at high risk of death within five years while minimizing false alarms that could lead to overtreatment.

Our analysis reveals a nuanced relationship between tumor location, sample size, and predictive performance that has important implications for clinical deployment (Supplementary Materials, Section S1.1, Table S1 and Figure S2). The superior performance in sigmoid colon cases (*n* = 149; AUC: 0.77 ± 0.06) compared to smaller anatomical subgroups suggests that both adequate sample representation and location-specific morphological patterns contribute to model reliability. The poor performance in hepatic flexure cases (*n* = 28; AUC: 0.56 ± 0.30) likely reflects both limited training examples and potentially distinct biological characteristics of tumors arising in different anatomical locations. These findings highlight the need for location-stratified model development or the incorporation of anatomical location as an explicit feature in future iterations. From a practical standpoint, this suggests that initial clinical deployment might prioritize the most common tumor locations where adequate training data exists, with subsequent expansion as larger datasets become available for rarer anatomical sites.

The robustness of PRISM across diverse clinical subgroups strengthens its translational potential. The minimal performance variation across sex subgroups (AUC difference of only 0.03 between male and female patients; Supplementary Materials, Section S1.2 and Table S2) and consistent performance across histological grades demonstrate that the model captures fundamental biological features rather than demographic artifacts (Table S3). Similarly, reproducing the Alliance cohort clinical trial results, with only 1.44% accuracy difference between FL and IFL regimens, aligns with clinical trial evidence showing equivalent survival outcomes and suggests that our model learns treatment-independent morphological features. We have also shown that improvement effect of integrating clinical attributes into account for existing baselines in Figures S3 and S4, and Tables S4 and S5.

### 5.1. Limitations and Challenges

Our study introduces an interpretable AI prognostic model that incorporates morphology-informed features for five-year survival prediction and addresses a critical gap in the interpretability of computational pathology models. PRISM’s interpretability stems from its ability to capture the continuous variability spectrum within distinct morphological patterns. This enables clinicians to understand the biological basis underlying model predictions, as each extracted feature correlates with specific tissue morphologies that pathologists can visually validate.

The interpretable nature of our morphology-informed features provides insights into how patient subgroups differ at the time of surgical resection, potentially serving as prognostic biomarkers for survival prediction. These morphological signatures reflect underlying tumor biology, micro-environmental characteristics, and disease progression patterns that influence long-term outcomes. In comparison to multi-omics approaches in immunotherapy and survival prediction: PRISM predicts the OS from routine H&E WSIs, potentially minimizing the need for costly, time-consuming molecular assays such as RNA sequencing, proteomics, or epigenomic profiling. Since PRISM is inherently interpretable, it allows for the identification of prognostically relevant phenotypes and may serve as a bridge between morphological phenotypes and underlying biological pathways, potentially paving the way to identify causal relationships. However, in its current form, there are several limitations that constrain PRISM’s ability to establish definitive causal relationships between resection-time morphological features and survival outcomes. This is because there is a temporal disconnect between patient surgical resection, treatment administration, and molecular-level information that multi-omics methods provide such as: immune gene expression signatures (e.g., interferon-γ pathway activity, cytotoxic T-cell transcriptional programs), tumor mutational burden, or detailed tumor microenvironment profiling. Since surgical resection precedes extended adjuvant therapy protocols, the morphological features captured at resection may not fully account for treatment-related biological changes that occur over the subsequent months to years. This temporal gap introduces uncertainty regarding whether the identified morphological patterns represent features that may be modified by subsequent therapeutic interventions.

Several limitations warrant consideration. First, our study focuses exclusively on stage III CRC patients from a single clinical trial, which limits generalizability to other stages and practice settings. The Alliance cohort [45], while well-characterized, represents a selected population that met specific trial inclusion criteria that may differ from routine clinical practice populations. Second, the morphology-aware classifier was trained on the Hist-AI colorectal dataset, which, while comprehensive, may not capture all morphological variations encountered in diverse clinical settings. Future work should explore training on larger, more diverse morphological datasets to enhance feature extraction capabilities. Third, we acknowledge that while treating survival prediction as a binary classification problem does not capture event timing information that Cox models provide, it offers direct answers to the specific clinical question “What is this patient’s probability of being alive in 5 years’ time?”, with interpretable performance metrics that enable unambiguous clinical assessments for treatment planning and surveillance strategies. Lastly, to establish robust interpretable biomarkers, future investigations require a more comprehensive data collection approach. This would necessitate molecular profiling at multiple timepoints throughout the treatment continuum, enabling researchers to distinguish between morphological features that remain stable predictors versus those that evolve with treatment response. Such holistic data collection strategies would strengthen the foundation for developing truly interpretable and clinically actionable prognostic biomarkers in CRC.

### 5.2. Broader Impact and Future Work

The integration of morphology-aware, high-level AI features with conventional histopathological characteristics represents a paradigm shift toward more sophisticated, biologically informed AI systems in oncology. Our AI prognostic model moves beyond the current limitations of “black box” deep learning models by incorporating domain-specific knowledge in a principled manner. The success of our AI prognostic model suggests broader applications across other cancer types where morphological heterogeneity plays a critical role in prognosis. The rigorous bias detection and mitigation strategies developed in this study provide a template for responsible AI development in healthcare. As AI systems become increasingly integrated into clinical workflows, the proactive identification and correction of algorithmic biases will be essential for ensuring equitable healthcare delivery.

Overall, PRISM presents an advancement in AI-driven CRC prognostication, demonstrating that morphology-aware, high-level feature extraction can substantially improve survival prediction accuracy. Beyond its technical contributions, this study establishes new standards for model validation and bias mitigation in clinical AI applications. PRISM’s robust performance across diverse patient subgroups, combined with its principled approach to addressing algorithmic bias, positions it as a promising tool for clinical translation. Future work will focus on extension to other cancer stages and types, and continued refinement of bias mitigation strategies to ensure equitable deployment across diverse patient populations.

## 6. Conclusions

This study presents PRISM, an AI prognostic model that demonstrates five-year overall survival prediction in stage III colorectal cancer from routine H&E WSIs. PRISM integrates domain-specific morphological features with general-purpose foundation model embeddings through a multi-instance learning framework and captures prognostic signals that conventional approaches overlook. Across five-fold cross-validation on a well-characterized clinical trial cohort, PRISM consistently outperformed existing CRC-specific and state-of-the-art AI methods, achieving superior AUC, accuracy, and hazard ratio with balanced sensitivity and specificity. Its robust performance across sex, tumor grade, treatment regimen, and anatomical subgroups underscores its generalizability. These results establish PRISM as a promising, interpretable tool for AI-driven prognostication, with potential for future extension to other cancer types and stages.

## Figures and Tables

**Table 1:** Demographic characteristics of the Alliance cohort.

Alliance Cohort Characteristics		
Number of slides		431
Number of Patients		424
Mean age (years)		60.47
Mean of household income median (USD)		43,194.59
Race	White	398
	Black	33
Sex	Male	240
	Female	191
Treatment	5FU/LV	219
	CPT-11/5FU/LV	212
Zubrod Performance scale	0	328
	1	98
	2	2
Tumor location	Cecum	101
	Ascending Colon	64
	Hepatic Flexure	28
	Transverse Colon	46
	Splenic flexure	19
	Descending colon	19
	Sigmoid colon	149
Grade	I	20
	II	300
	III	108
	IV	0
Stage	I	6
	II	42
	III	350
	IV	8
	V	20
Small blood/ lymphatic vessel invasion	No	285
	Yes	138
Extramural vascular invasion	No	387
	Yes	29
Infiltrating border	No	274
	Yes	141
Five-year survival	Yes	29
	Survived	328

**Table 2:** Five-year survival prediction results of PRISM stratified by FL/IFL treatments in the Alliance cohort using five-fold cross-validation. Each row reports the average performance with standard deviation.

Model	Treatment	AUC	Accuracy (%)	Sensitivity (%)	Specificity (%)
ABMIL [39]	FL	0.65 ± 0.11	62.02± 13.76	51.93 ± 22.92	72.12± 08.70
	IFL	0.58 ± 0.07	54.13± 05.61	45.34 ± 08.90	62.92± 06.89
CLAM [35]	FL	0.61 ± 0.09	50.04± 05.96	14.12± 08.97	85.96 ± 08.47
	IFL	0.63 ± 0.15	60.89± 06.01	36.71± 13.97	85.07 ± 09.81
TransMIL [40]	FL	0.75± 0.10	54.75± 08.37	10.33 ± 16.13	99.16± 01.66
	IFL	0.62± 0.09	51.81± 03.63	3.63 ± 07.27	100.00± 00.00
Nakanishi et al. [31]	FL	0.65 ± 0.16	61.12± 12.85	48.38± 21.23	73.85± 07.18
	IFL	0.55 ± 0.07	56.02± 05.29	56.23± 10.32	55.80± 08.49
RRT-MIL [41]	FL	0.59± 0.15	55.42 ± 08.54	43.45 ± 25.65	67.38 ± 25.52
	IFL	0.55± 0.04	55.62 ± 03.61	46.90 ± 24.09	64.35 ± 25.62
PRISM	FL	0.72 ± 0.06	68.21 ± 03.40	64.82 ± 03.40	71.60 ± 08.50
	IFL	0.68 ± 0.11	66.77 ± 05.10	68.76 ± 13.90	64.77 ± 10.90

## Data Availability

This study used the publicly available TCGA-COAD, TCGA-READ dataset, which can be accessed at https://portal.gdc.cancer.gov/projects/TCGA-READ (accessed on 1 May 2025), https://portal.gdc.cancer.gov/projects/TCGA-COAD (accessed on 1 May 2025); https://huggingface.co/datasets/histai/SPIDER-colorectal (accessed on 1 February 2025). The data from CALGB 89803 were obtained directly from the Alliance for Clinical Trials in Oncology, a National Clinical Trials Network cooperative group; It can be accessed by contacting Alliance and signing an agreement with them. All the relevant dataset references have been cited within the manuscript. All analyses and conclusions in this manuscript are the sole responsibility of the authors and do not necessarily reflect the opinions or views of the clinical trial investigators, the NCTN, the NCORP or the NCI. The underlying code for this study is available in AI4Path PRISM repository and can be accessed via following link: https://github.com/AI4Path-Lab/PRISM (accessed on 1 May 2025).

## Supplementary Materials

The following supporting information can be downloaded at https://www.mdpi.com/article/10.3390/cancers18071150/s1, Figure S1: An overview of our PRISM framework. Figure S2: Subgroup analysis of our model’s performance stratified by colon tumor location in the Alliance cohort using five-fold cross-validation. Figure S3: Five-year survival prediction results in the Alliance cohort using our proposed five-fold cross-validation method. Figure S4: Hazard ratios and concordance index (c-index) values in the Alliance cohort using our proposed validation strategy. Table S1: Five-year OS results of PRISM stratified by tumor location in the Alliance cohort using five-fold cross-validation. Table S2: Five-year survival prediction results of PRISM stratified by sex in the Alliance cohort using five-fold cross-validation. Table S3: Five-year survival prediction results of PRISM stratified by Grade in the Alliance cohort using five-fold cross-validation. Table S4: Five-year survival prediction results of PRISM stratified by FL/IFL treatments in the Alliance cohort using our proposed validation strategy. Table S5: Five-year survival prediction results of PRISM stratified by sex in the Alliance cohort using our proposed validation strategy. Table S6: c-index values got using TCGA-COADREAD dataset. All methods were trained using five-fold cross validation.
