# Supplementary material for: Morphology-Aware Prognostic Model for Five-Year Survival Prediction in Colorectal Cancer from H&E Whole-Slide Images: A Study Using Multi-Center Clinical Trial Cohort
Source: Cancers (Basel). 2026 Apr 2;18(7):1150. doi: 10.3390/cancers18071150 (PMC13072141; doi:10.3390/cancers18071150)
Supplement: Supplementary file 1 [file cancers-18-01150-s001.zip › cancers-4194057-supplementary.pdf]

Architecture:

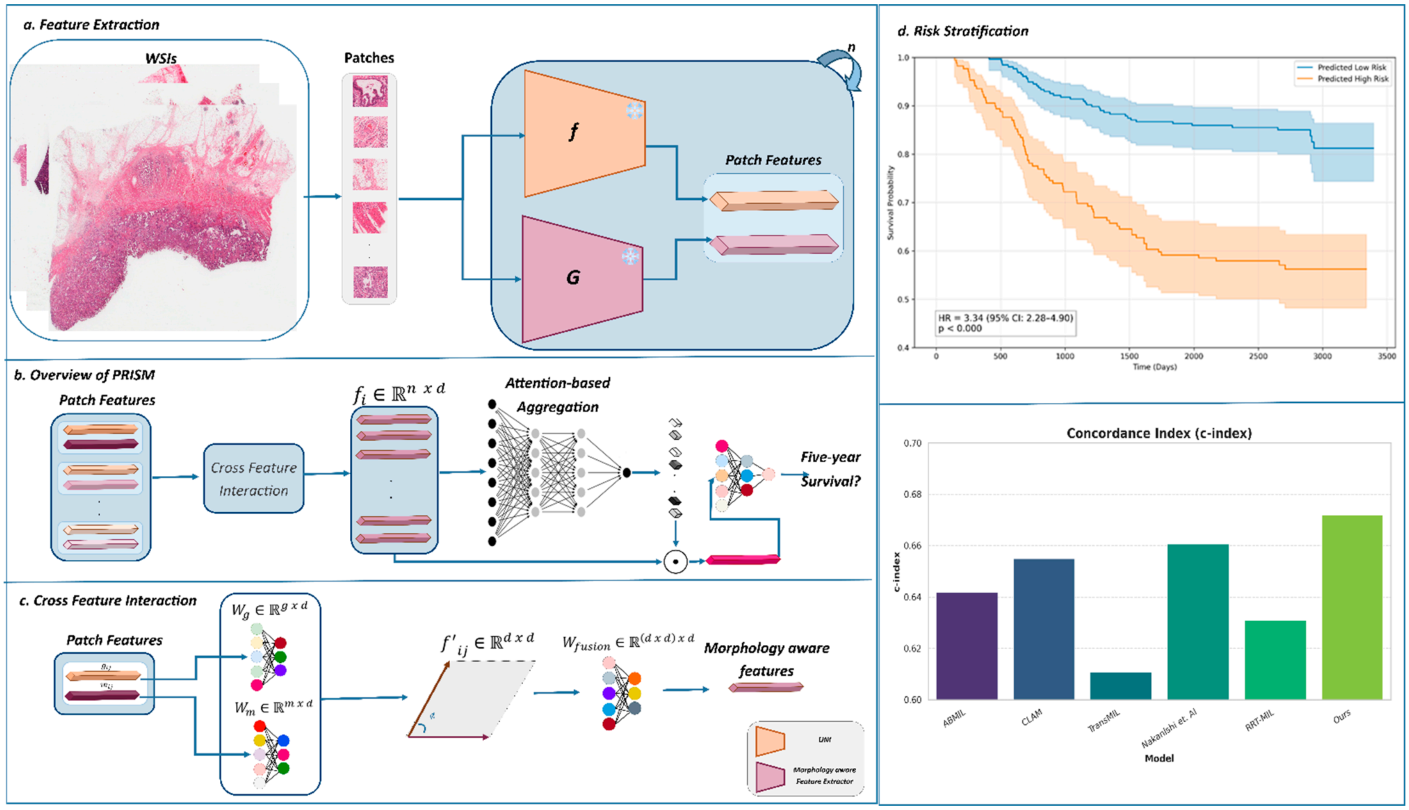

Figure S1: An overview of our PRISM framework. (**a**) We first tessellate WSIs into n non-overlapping patches, with each patch undergoing dual feature extraction. (**b**) We perform cross-feature interaction between universal pathology features from UNI and morphology-aware features that encode tissue architecture and histopathological patterns. We then fuse these complementary feature representations $f_{i,j}$ at the patch level to create comprehensive morphological embeddings. An attention mechanism computes importance scores for each patch feature $f_{i,j}$ based on its prognostic relevance, enabling the model to focus on histologically relevant regions. We aggregate attention-weighted patch embeddings into a slide-level representation that captures the overall morphological landscape for five-year survival prediction. (**c**) During patch feature aggregation, we project features using two different neural networks (W_g, W_m) and aggregate the results to obtain morphology-aware features for each patch using W_{fusion}. (**d**) Based on the predicted probability, we train a time-to-event Cox Hazards model or perform risk stratification using concordance index.

S1. Additional Results:

S1.1. Tumor Location-Dependent Survival Prediction Using PRISM: Larger Patient Numbers Offset Batch Effects and Enhance Performance

To assess the generalizability and clinical applicability of PRISM across different colorectal anatomical sites, we conducted comprehensive subgroup analysis stratified by tumor resection location. This evaluation is critical for understanding how morphological feature patterns and model performance vary across the diverse biological microenvironments within the colon, informing clinical deployment strategies and identifying potential limitations in site-specific prognostication.

The prognostic performance across colon tumor resection cohorts exhibited a strong correlation with total sample size (n), where larger patient groups stratified by tumor location yield more reliable prognostic performance, though inherent biological differences in feature patterns across anatomical locations may also contribute to performance variations. As detailed in Table S1 and Figure S2, in patients with sigmoid colon cancers (n=149), PRISM achieved optimal performance (AUC: 0.77 ± 0.06; C-index: 0.75; HR: 3.88). Following this, in cecal cancers (n=101), PRISM demonstrated moderate but stable metrics (AUC: 0.71 ± 0.11; C-index: 0.69; HR: 2.97), showing the performance drop despite adequate sampling. This performance decline accelerated with ascending colon cancers (n=64), where results remained robust (AUC: 0.74 ± 0.21; C-index: 0.71; HR: 3.10) but exhibited high standard deviation. In transverse colon cancers (n=46), PRISM demonstrated

high sensitivity (84.52 ± 15.5) but critically low specificity (50.01 ± 17.46), suggesting location-specific feature importance may skew predictions. Conversely, smaller cohorts revealed significant limitations: splenic flexure cancers (n=19) had the poorest AUC (0.42 ± 0.43) and C-index (0.49), while hepatic flexure (n=28) and descending colon cancers (n=19) showed near-random sensitivity (50.0 ± 50.0) and marginal hazard ratios (3.29 and 2.39) with large 95% confidence intervals (Figure S2), indicating that both limited samples and possibly local specific features may compromise model generalizability. These results collectively demonstrate that reliability of deep learning models depends critically on cohort size, and tumor microenvironment differences across locations, necessitating location-specific feature extraction for clinically viable models.

Table S1: Five-year OS results of PRISM stratified by tumor location in the Alliance cohort using five-fold cross-validation. Each row reports the average performance with standard deviation. For each metric, the best result is shown in bold, and the second best is underlined.

	Tumor Location	AUC	Accuracy (%)	Sensitivity (%)	Specificity (%)	No of Samples (n)
**Right Colon**	Cecum	0.71 ± 0.11	67.58 ± 6.70	67.85 ± 0.20	67.32 ± 11.00	101
	Ascending Colon	0.74 ± 0.21	**72.46 ± 20.32**	74.28 ± 0.24	70.64 ± 17.39	64
	Hepatic Flexure	0.56 ± 0.30	60.77 ± 22.22	60.95 ± 25.71	60.60 ± 22.81	28
	Transverse Colon	0.74 ± 0.19	67.26 ± 3.80	**84.52 ± 15.5**	50.01 ± 17.46	46
	Right Colon	0.69 ± 0.05	68.08 ± 3.59	**71.62 ± 10.12**	64.53 ± 10.56	239
**Left Colon**	Splenic Flexure	0.42 ± 0.43	65.83 ± 15.87	50.00 ± 50.00	81.66 ± 18.48	19
	Descending Colon	0.46 ± 0.27	64.58 ± 14.87	50.00 ± 50.00	**79.16 ± 21.65**	19
	Sigmoid Colon	**0.77 ± 0.06**	**70.09 ± 3.70**	64.69 ± 8.40	75.49 ± 12.29	**149**
	Left Colon	0.71 ± 0.08	67.69 ± 5.69	**61.39 ± 12.66**	73.99 ± 10.03	187

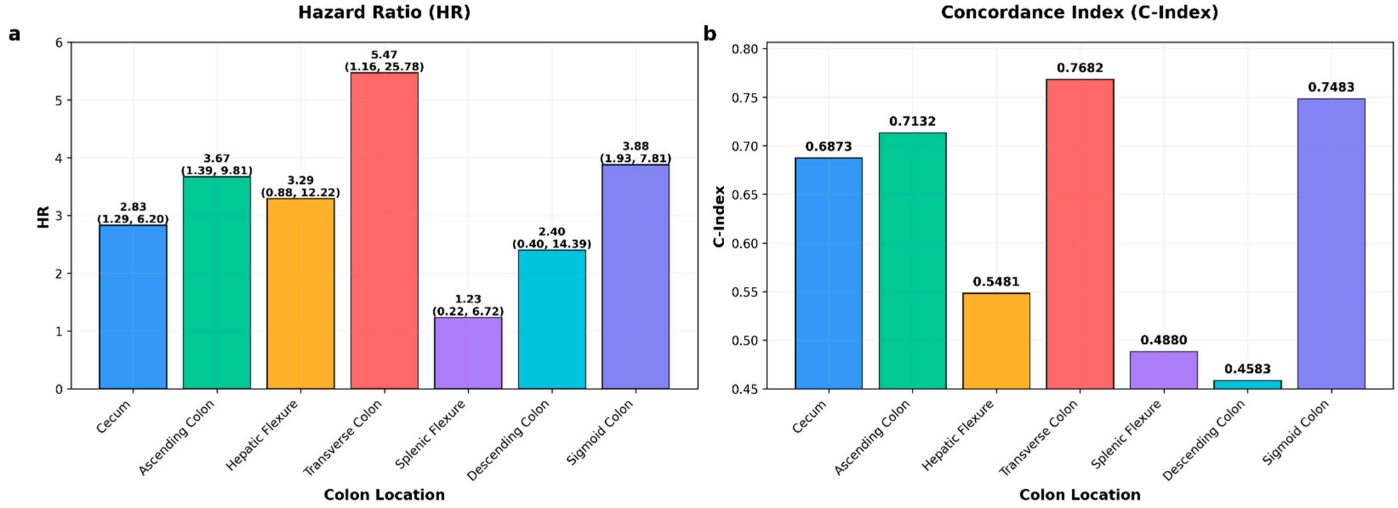

Figure S2: Subgroup analysis of our model's performance stratified by colon tumor location in the Alliance cohort using five-fold cross-validation. (**a**) Hazard ratios with 95% confidence intervals for each anatomical site, and (**b**) Concordance Index (C-Index) values demonstrating model's ability to risk-stratify patients across different colon segments. PRISM achieved the highest C-Index (0.7483) and robust hazard ratio (3.88) in sigmoid colon cancers, while smaller cohorts from splenic flexure and descending colon showed reduced performance, indicating that model reliability correlates with sample size and possibly anatomical site-specific biological features.

S1.2. Robustness of PRISM Across Demographics and Histology

A critical requirement for clinical deployment of AI-based prognostic models is demonstrated robustness across diverse populations and clinical contexts. To address concerns about generalization and bias, we conducted comprehensive subgroup analyses of PRISM within the Alliance cohort, evaluating performance consistency across demographic and pathological stratifications. These analyses confirm PRISM's superior stability and reliability compared to benchmark methods (ABMIL, CLAM, Nakanishi et al., RRT-MIL).

PRISM exhibited strong consistency across sexes (Table S2), a key indicator of clinical robustness. Performance metrics remained stable between male (AUC: 0.69 ± 0.06; accuracy: 68.06% ± 5.00) and female (AUC: 0.71 ± 0.08; accuracy: 67.91% ± 3.20) cohorts, with minimal AUC variation (Δ = 0.02) and negligible accuracy fluctuation (0.15% difference). In contrast, benchmarks showed significant sex-based instability: CLAM had a 5.5% accuracy drop between sexes, while Nakanishi et al. and RRT-MIL exhibited 1.5–2% accuracy reductions and extreme sensitivity variability (e.g., RRT-MIL sensitivity SD: ±30.91). This consistency underscores PRISM's resilience to sex-based distribution shifts, suggesting reliance on generalizable biological features rather than spurious correlations.

Notably, PRISM achieved superior performance in female patients (highest AUC: 0.71 ± 0.078; highest accuracy: 67.91% ± 3.20), surpassing CLAM by 15% in accuracy while delivering better-balanced sensitivity/specificity (64.00% ± 11.0/71.81% ± 10.00 vs. 20.33% ± 12.13/85.16% ± 13.61) (Table S2). Its higher sensitivity is clinically critical for identifying high-risk female patients requiring aggressive intervention. In male patients, PRISM outperformed benchmarks in practical utility: while TransMIL had a marginally higher AUC (0.67 ± 0.06), its AUC is influenced by predicting majority of the samples as five-year survivors, whereas PRISM achieved ~8% higher accuracy (68.06% ± 5.00) with balanced sensitivity/specificity (69.7% ± 9.00/66.58% ± 11.00) and lower metric variability, avoiding trade-offs observed in competitors.

Table S2. Five-year survival prediction results of PRISM stratified by sex in the Alliance cohort using five-fold cross-validation. Each row reports the average performance with standard deviation. For each metric, the best result is shown in bold, and the second-best is underlined.

Model	Sex	AUC	Accuracy (%)	Sensitivity (%)	Specificity (%)
ABMIL [29]	Female	0.63 ± 0.11	57.86 ± 10.13	51.48 ± 17.68	64.25 ± 07.50
	Male	0.61 ± 0.05	57.68 ± 05.75	46.04 ± 12.87	69.32 ± 07.46
CLAM [1]	Female	0.61 ± 0.16	52.75 ± 09.29	20.33 ± 12.13	85.16 ± 13.61
	Male	0.63 ± 0.03	58.35 ± 03.95	31.38 ± 08.05	85.33 ± 05.95
TransMIL [30]	Female	0.69 ± 0.12	54.18 ± 05.99	08.37± 11.99	01.00 ± 00.00
	Male	0.67 ± 0.06	52.67 ± 06.19	06.00 ± 12.00	99.35 ± 01.20
Nakanishi et. Al [32]	Female	0.60 ± 0.11	58.32 ± 09.70	53.43 ± 20.25	63.21 ± 07.87
	Male	0.61 ± 0.06	59.99 ± 05.78	56.01 ± 12.59	63.97 ± 03.65
RRT-MIL [31]	Female	0.56 ± 0.07	54.11 ± 05.35	45.98 ± 30.91	62.24 ± 24.09
	Male	0.59 ± 0.08	56.17 ± 06.29	44.19 ± 21.72	68.16 ± 25.79
PRISM	Female	0.71 ± 0.07	67.91± 03.20	64.00 ± 11.00	71.81 ± 10.00
	Male	0.69 ± 0.06	68.06 ± 05.00	69.70 ± 09.00	66.38 ± 11.00

PRISM also generalized robustly across histologic heterogeneity (Table S3). Despite known inter-observer variability in grading, performance remained consistent across glandular differentiation grades:

- **Grade I** (well differentiated, n=20): High accuracy (81.25% ± 6.25) and AUC (0.75 ± 0.25), though variability reflected sample limitations.
- **Grade II** (moderately differentiated, n=297): Stable accuracy (67.73% ± 3.31) and AUC (0.71 ± 0.03) with low standard deviation.
- **Grade III** (poorly differentiated, n=104): Robust accuracy (67.25 ± 4.72) and AUC (0.70 ± 0.10)

This consistency across biologically distinct grades—particularly the stability in Grade II (the largest subgroup)—demonstrates PRISM's capacity to learn generalizable pathologic features. PRISM establishes a new standard for robust prognostication, consistently matching or outperforming benchmarks across genders and histologic grades while avoiding sensitivity-specificity trade-offs. Its minimal performance fluctuation in key subgroups (ΔAUC <0.03 across sexes; <4% accuracy variability in Grade II and III), superior accuracy in males and leading female-cohort performance in comparison with comparison methods, and adaptability to histologic heterogeneity directly address clinical concerns about generalization and bias. These attributes position PRISM as a uniquely reliable model for clinical translation in CRC prognosis.

Table S3. Five-year survival prediction results of PRISM stratified by Grade in the Alliance cohort using five-fold cross-validation. Each row reports the average performance with standard deviation. For each metric, the best result is shown in bold, and the second-best is underlined.

Grade	AUC	Accuracy (%)	Sensitivity (%)	Specificity (%)	No of patients (n)
I	0.75 ± 0.25	81.25 ± 6.25	75.00 ± 25.00	87.50 ± 12.5	20
II	0.71 ± 0.03	67.73 ± 3.31	65.11 ± 6.92	70.35 ± 8.99	297
III	0.70 ± 0.10	67.25 ± 4.72	72.85 ± 18.90	61.64 ± 14.63	104

S1.3. Enhanced Performance of Baseline Methods using Add-on Module Integration and PRISM Superiority

To rigorously evaluate the impact of our proposed patient heterogeneity module, we applied this add-on enhancement to existing state-of-the-art methods and compared their performance against PRISM. The integration of our module consistently improved the prognostic accuracy of baseline models across multiple clinical subgroups, though PRISM maintained superior performance in all comparisons.

When applied to CLAM, our add-on module substantially improved its five-year survival prediction accuracy by 7% compared to baseline, ensuring that model training incorporates clinically relevant subgroups (Supplementary Figure S3). In the FL treatment subgroup, CLAM's accuracy increased from 50.04 ± 5.96% to 63.29 ± 4.45%, representing a 13.25% absolute improvement and reduced variability (Supplementary Table S4). Similarly, in female patients, CLAM's accuracy improved from 52.75 ± 9.29% to 65.10 ± 8.70% (a 12.35% gain) (Supplementary Table S5). However, these enhancements did not surpass PRISM's performance, which achieved an accuracy of 68.21 ± 3.40% in the FL subgroup and 67.91 ± 3.20% in females, representing a 4.92% and 2.81% improvement, respectively. ABMIL also demonstrated notable gains with our module. For FL-treated patients, accuracy improved from 62.02 ± 13.76% to 63.86 ± 6.30, a 1.84% increase with significantly reduced standard deviation (from 13.76% to 6.30%). Sex-stratified analysis also revealed an 8% accuracy improvement in female patients (from 57.86 ± 10.13% to 61.71 % ± 8.20). Despite these improvements, PRISM still outperformed enhanced ABMIL by 4.35% in the FL subgroup and 8.77% in the IFL subgroup. Nakanishi et al.'s method showed more modest gains, with FL treatment accuracy improving from 61.12 ± 12.85 to 62.21 ± 4.70 and IFL accuracy increasing from 56.02 ± 5.29 to 58.83 ± 8.60. RRT-MIL exhibited variable performance: FL accuracy improved from 55.42

± 8.54 to 59.51 ± 11.11 (a 4.09% gain), but IFL accuracy decreased from 55.62 ± 3.61 to 51.18 ± 9.42 (a 4.44% drop), indicating instability.

The HR analysis further validated PRISM's dominance, achieving a robust HR of 3.34 (95% CI: 2.28–4.90)—1.5× greater risk discrimination than the state-of-the-art methods (Supplementary Figure S4). While CLAM improved marginally (HR: 1.96 → 2.25) but failed to approach PRISM's precision, as their underlying architectures could not fully leverage the continuous morphological spectrum essential for robust risk stratification. PRISM's tight confidence interval (spanning 2.62 vs. TransMIL's 9.98) enabled clinically actionable identification of high-risk patients with 3.34× greater mortality likelihood within five years.

Critically, the add-on module reduced performance variability across all baselines, as evidenced by lower standard deviations in key subgroups (Supplementary Table S4). For instance: CLAM's standard deviation in the FL subgroup decreased from ±5.96% to ±4.45%. ABMIL's FL subgroup variability dropped sharply from ±13.76 to ± 6.30%. Nakanishi et al.'s FL subgroup variability reduced from ±12.85 to ± 4.70%. This confirms that accounting for population heterogeneity mitigates algorithmic bias and enhances model stability.

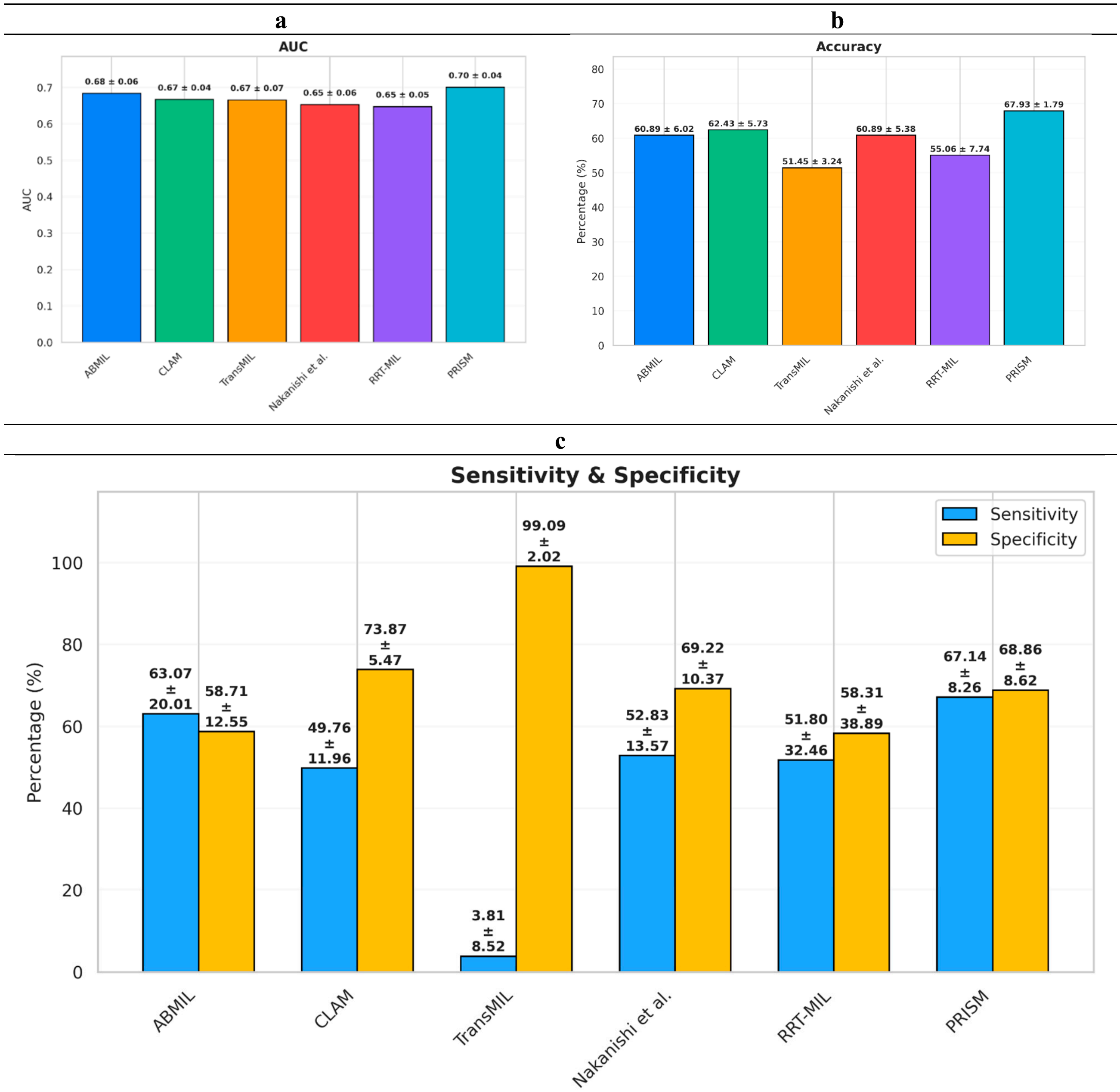

Figure S3. Five-year survival prediction results in the Alliance cohort using our proposed five-fold cross-validation method. (**a**) Area Under the Curve (AUC) values with standard deviations, (**b**) Accuracy percentages with standard deviations, and (**c**) Grouped

comparison of sensitivity and specificity with other models. Our model achieves the highest accuracy and balanced sensitivity and specificity, demonstrating superior performance compared to existing state-of-the-art methods.

Table S4. Five-year survival prediction results of PRISM stratified by FL/IFL treatments in the Alliance cohort using our proposed validation strategy. Each row reports the average performance with standard deviation. For each metric within a treatment, the best result is shown in bold, and the second best is underlined.

Model	Treatment	AUC	Accuracy (%)	Sensitivity (%)	Specificity (%)
ABMIL [30]	FL	0.7118 ± 0.058	63.86 ± 6.30	65.26 ± 17.11	62.46 ± 12.28
	IFL	0.6576 ± 0.141	58.00 ± 10.85	61.76 ± 27.66	54.24 ± 15.40
CLAM [26]	FL	0.6820 ± 0.044	63.29 ± 4.45	48.63 ± 9.40	77.96 ± 7.96
	IFL	0.6444 ± 0.112	59.85 ± 11.17	49.62 ± 22.04	70.09 ± 3.92
Transmil [31]	FL	0.7132 ± 0.057	51.89 ± 3.70	4.40 ± 8.80	99.35 ± 1.29
	IFL	0.6091 ± 0.098	51.09 ± 2.10	3.30 ± 6.60	98.80 ± 2.20
Nakanishi et. Al [33]	FL	0.6914 ± 0.080	62.21 ± 4.70	56.24 ± 17.12	68.18 ± 11.43
	IFL	0.6843 ± 0.128	58.83 ± 8.60	48.16 ± 15.27	69.51 ± 15.43
RRT-MIL [32]	FL	0.6961 ± 0.036	59.51 ± 11.11	59.82 ± 32.59	59.19 ± 36.46
	IFL	0.5962 ± 0.159	51.18 ± 9.42	46.30 ± 33.37	56.07 ± 42.83
PRISM	FL	0.7160 ± 0.061	68.21 ± 3.40	64.82 ± 3.40	71.60 ± 8.50
	IFL	0.6846 ± 0.108	66.77 ± 5.10	68.76 ± 13.90	64.77 ± 10.90

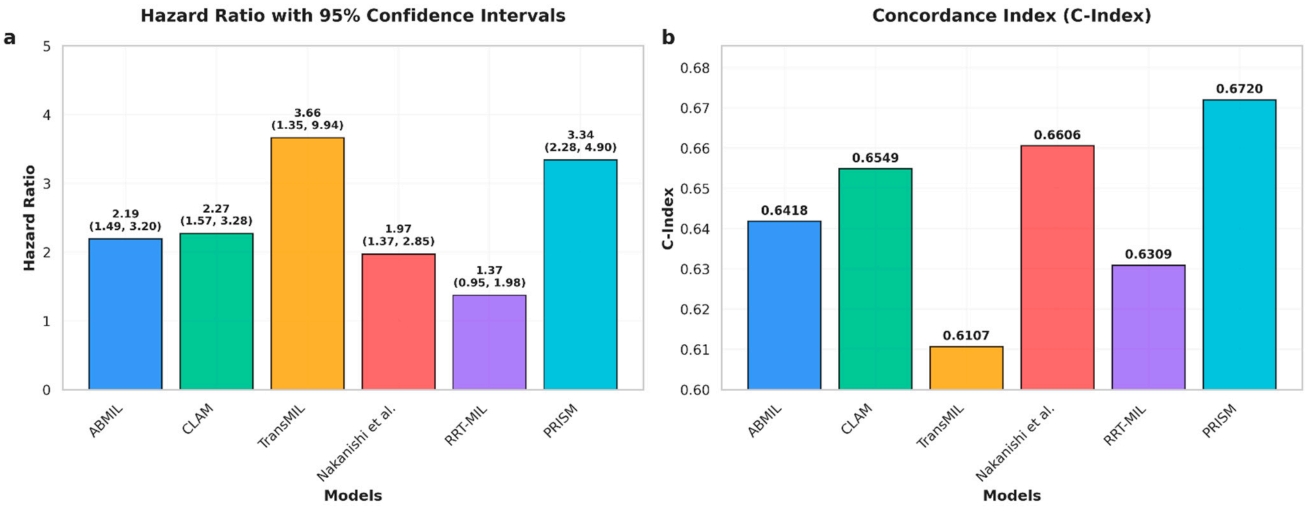

Figure S4. Hazard ratios and concordance index (c-index) values in the Alliance cohort using our proposed validation strategy. (a) Hazard ratios with 95% confidence intervals for each model, and (b) Concordance Index (C-Index) values demonstrating model ability to sort the patient based on risk for survival prediction. Our model achieves the highest C-Index (0.6720) and comparable hazard ratio in comparison with TransMIL with smaller confidence interval, indicating superior prognostic performance compared to other state-of-the-art methods.

Table S5. Five-year survival prediction results of PRISM stratified by sex in the Alliance cohort using our proposed validation strategy. Each row reports the average performance with standard deviation. For each metric, the best result is shown in bold, and the second-best is underlined.

Model	Sex	AUC	Accuracy (%)	Sensitivity (%)	Specificity (%)
ABMIL [39]	Female	0.6816 ± 0.079	61.71 ± 8.20	60.87 ± 19.10	62.54 ± 13.14
	Male	0.6906 ± 0.064	59.26 ± 5.10	64.51 ± 22.47	54.73 ± 15.35
CLAM [35]	Female	0.7046 ± 0.070	65.10 ± 8.70	56.34 ± 16.78	73.85 ± 8.72
	Male	0.6377 ± 0.053	59.90 ± 5.50	45.87 ± 13.08	73.93 ± 5.80
Transmil [40]	Female	0.6699 ± 0.110	52.85 ± 5.70	5.70± 11.42	1.0 ± 0.0
	Male	0.6541 ± 0.054	50.57 ± 1.10	2.80 ± 5.70	98.28 ± 3.40
Nakanishi et. Al [31]	Female	0.6796 ± 0.080	59.33 ± 6.20	46.70 ± 9.60	71.96 ± 14.18
	Male	0.6672 ± 0.043	59.96 ± 2.60	54.05 ± 14.40	65.86 ± 13.73
RRT-MIL [41]	Female	0.6299 ± 0.055	55.95 ± 7.08	52.46 ± 33.69	59.45 ± 37.99
	Male	0.6535 ± 0.056	54.37 ± 8.67	51.38 ± 31.69	57.36 ± 40.05
PRISM	Female	0.7120 ± 0.07	67.91± 3.20	64.00 ± 11.0	71.81 ± 10.00
	Male	0.6869 ± 0.060	68.06 ± 5.00	69.7 ± 9.00	66.38 ± 11.00

Table S6. c-index values got using TCGA-COADREAD dataset. All methods were trained using five-fold cross validation.

Model	C-index
ABMIL	0.6034 ± 0.0500
PANTHER	0.5832 ± 0.0735
DSMIL	0.5 ± 0.0
RRT-MIL	0.5991 ± 0.08094
**PRISM**	**0.6268 ± 0.0612**
